# Impact of a standardized training program on midwives’ ability to assess fetal heart anatomy by ultrasound

**DOI:** 10.1186/1471-2342-14-20

**Published:** 2014-06-02

**Authors:** Eric Hildebrand, Madeleine Abrandt Dahlgren, Catarina Sved, Tomas Gottvall, Marie Blomberg, Birgitta Janerot-Sjoberg

**Affiliations:** 1 Department of Obstetrics and Gynaecology, and Department of Clinical and Experimental Medicine, Linköping University, Linköping, Sweden; 2 Department of Medicine and Health Sciences, Faculty of Health Sciences, Linköping University, Linköping, Sweden; 3 Department of Clinical Physiology and Nuclear Medicine, University Hospital, Linköping, Sweden; 4 Department of Medicine & Health, Division of Cardiovascular medicine, Faculty of Health Sciences, Linköping University, Linköping, Sweden; 5 Department Biomedical Engineering, Linköping University, Linköping, Sweden; 6 Department of Clinical Physiology, Karolinska University Hospital, Stockholm, Sweden; 7 Department of Clinical Science, Division of Medical Imaging and Technology, Intervention and Technology, Karolinska Institutet, Stockholm, Sweden

**Keywords:** Color Doppler, Congenital heart disease, Detection of congenital heart defects, Fetal heart scanning, Learning program, Prenatal cardiology, Second trimester screening, Standardized training program, Ultrasound screening

## Abstract

**Background:**

Studies of prenatal detection of congenital heart disease (CHD) in the UK, Italy, and Norway indicate that it should be possible to improve the prenatal detection rate of CHD in Sweden. These studies have shown that training programs, visualization of the outflow tracts and color-Doppler all can help to speed up and improve the detection rate and accuracy. We aimed to introduce a more accurate standardized fetal cardiac ultrasound screening protocol in Sweden.

**Methods:**

A novel pedagogical model for training midwives in standardized cardiac imaging was developed, a model using a think-aloud analysis during a pre- and post-course test and a subsequent group reflection. The self-estimated difficulties and knowledge gaps of two experienced and two beginner midwives were identified. A two-day course with mixed lectures, demonstrations and hands-on sessions was followed by a feedback session three months later consisting of an interview and check-up. The long-term effects were tested two years later.

**Results:**

At the post-course test the self-assessed uncertainty was lower than at the pre-course test. The qualitative evaluation showed that the color Doppler images were difficult to interpret, but the training seems to have improved their ability to use the new technique. The ability to perform the method remained at the new level at follow-up both three months and two years later.

**Conclusions:**

Our results indicate that by implementing new imaging modalities and providing hands-on training, uncertainty can be reduced and examination time decreased, but they also show that continuous on-site training with clinical and technical back-up is important.

## Background

Congenital heart disease (CHD) is the most common congenital defect and can lead to major adverse consequences for the child. Internationally, just below 1% of all pregnancies are affected by CHD and usually there is no identifiable cause [1,2]. About half of the CHD cases are regarded as major, requiring surgery or intervention in the child’s first year of life. Furthermore, about one third of the major CHD cases will have a duct-dependent anomaly, an anomaly that, if not identified before birth or recognized shortly after birth, will become a life threatening condition [3,4].

In a recently published study, the incidence of CHD in the Southeast region of Sweden was 8/1,000 fetuses/newborns whereof 3/1,000 were major. The detection rate of CHD cases was only 5% before birth if all cases were included but became 37,5% if all cases of minor CHD were excluded [5]. Similar results were found in another large Swedish study (n = 36,299) where 15% of major CHDs were detected before 22 weeks [6]. A study from Trondheim in Norway reported a 46% detection rate of major CHD at the time of the 18 week routine scan [4]. Del Bianco et al. state that in Italy almost 90% of major CHD defects are detectable before birth if a full heart examination is performed at 20–24 weeks of gestation [7]. These data indicate that the Swedish prenatal detection rate of CHD has a potential for being significantly increased and that the screening performance is in need of improvement. Recognition of this situation was what led us to make this study.

Training programs have been proven effective in increasing the antenatal detection of CHD. McBrien et al. reported a rise in detection rate from 28% to 43% after a relatively simple training program was implemented in the UK [8]. Oggè et al. conducted a multicenter study in Italy, and after training the sensitivity for CHD was 65,5% with a specificity of 99,7% [9]. Allen et al. demonstrated a marked geographical difference in detection rate of structural cardiac defects in the UK, ranging from 0 to 70%, where the higher rates of detection tended to be concentrated in areas where teaching programs in cardiac scanning had been in place for some time [10]. Results from a study by Pézard et al. in France support the hypothesis that providing learning centers where screening sonographers may improve their practice will have beneficial effects [11].

In the Southeast region of Sweden all pregnant women are offered two routine ultrasound examinations as part of the official Maternity Health Care System, one at 11 to 14 gestational weeks (“first trimester”) in order to assess the gestational age, and another at 18 to 20 weeks (“second trimester”) to assess the anatomical features of the fetus. The screenings are performed by specially trained midwives and 30 minutes are allocated for each scan. In the first trimester screening all women are also offered a first-trimester combined risk-assessment for trisomy 21, 13 and 18 [12]. At the second trimester scan a checklist is used for anatomical assessments. Concerning the heart, the screening results are regarded as normal if the four-chamber view is accurately obtained. This criterion has been used in the screening-situation as a simple and reproducible basis for finding anomalies of the fetal heart. However, this approach has been shown to detect only a minority of heart anomalies; it does not identify anomalies affecting the outflow tracts and the great arteries [7,13]. Adding visualization of the outflow tracts to the four-chamber view has been proven to be an effective technique to detect major CHD prenatally [14]. Previous studies have shown that using a systematic approach when examining the fetal heart facilitates the confirmation of normality and also makes it easier to recognize abnormalities [10,13-16]. If color-Doppler is added to the examination, rapid screening to detect flow abnormalities of the fetal heart may be carried out [17]. This view is supported by Chaoui et al. who suggest that color-Doppler should be added to the screening for CHD in order to allow easy detection of the majority of CHD [18]. Results from a recent study by Eggebo et al. led the authors to conclude that the routine use of color Doppler in fetal heart scanning may be helpful in the detection of major CHD [19].

The aim of this study was to evaluate the possibility for introducing a more accurate fetal cardiac ultrasound screening method, based on five additional transverse views and color-Doppler, by using a novel pedagogical approach to the standardized cardiac imaging training.

## Methods

A two-day course in standardized examination techniques of the fetal heart and color-Doppler was given at Linköping University Hospital. The course was designed by a group consisting of physicians and researchers specialized in fetal medicine, pediatric cardiology and clinical physiology, a specialized sonographer, and a professor of education. Four midwives from the routine ultrasound screening in the southeast region of Sweden attended the course; two were from Linköping University Hospital and two from Värnamo county hospital. Each hospital contributed one experienced midwife with more than 20 years of experience with obstetrical ultrasound and one beginner with five years or less of experience. The local ultrasound units at the hospitals were free to choose the midwives to participate as long as they met these criteria and had given their informed consent. At arrival, a pre-course test was taken by the midwives. The intent of the pre-course test was to enhance the midwives’ awareness of their present level of knowledge and skills and to identify any difficulties encountered in using the new technique, as a starting point for learning. At this test, each midwife was individually presented a series of ultrasound recordings of the fetal heart visualized as cine-loops in gray-scale with or without color Doppler. The midwives were equipped with dictaphones and instructed to “think aloud” by saying how they judged the scans and what they based their judgment on [20]. They were also instructed to comment on the quality of the scans and to note if the color Doppler added significant information. In addition, the midwives filled out a paper form on which they indicated if they judged the scan to be normal or pathological and how confident they felt about the judgment. The experiences from this test were then discussed and the discussion was audio-recorded by the group of four and the course leaders. Thirty minutes were allocated for the discussion. The recording was intended to then serve as a basis for reflection and as a source indicating needs for improved knowledge and skills. Based on the results, a detailed course syllabus was created, setting forth a program that would consist of lectures on ultrasound examination of the fetal heart using color Doppler technique as well as hands-on training. The first step was for the midwives, in the presence of cardiac sonographers, to practice color-Doppler examination of children and adolescents who came to the Department of Clinical Physiology for cardiac ultrasound exams where color-Doppler is routinely used. Each midwife used two hours of training on different patients. Informed consent was given by the patients. On the second day of the course, fetal heart screening was performed on pregnant women who had reached about 20 weeks of gestation. Another individual two hours of training on different fetuses were allocated for each midwife after she had been given informed consent by each pregnant woman. For the clinical praxis the midwives were taught to record an ultrasound gray-scale-clip starting from the abdomen of the fetus and sliding in the cephalic direction; this procedure was repeated with color Doppler added. The different anatomical landmarks are described in Table 1.

**Table 1:** Protocol used for standardized examination of the fetal heart

1	Position of the fetus to determine the situs
2	Cross-section of the fetal abdomen, then slide in the cephalic direction
	S: Determine the position of the stomach, inferior vena cava and the aorta
	A: Four-chamber view
	B: Outflow-tract of aorta from left ventricle
	C: Outflow tract of pulmonary artery
	D: Three vessel view and arches
3	Repeat the slide with addition of color Doppler
	A: Four-chamber view
	B: Outflow-tract of aorta from left ventricle
	C: Outflow tract of pulmonary artery
	D: Three vessel view and arches

With these experiences a second thirty minute group discussion session was held and digitally audio-recorded for later analysis. The course ended by having the midwives take the same individual audio-recorded and form-completion test that they had taken before starting the course.

Before leaving, the midwives were given another protocol to be used in their clinical practice until first follow-up. This protocol included documentation of their self-assessment in a four-graded scale of their confidence in performing the different steps when examining the fetal heart (Table 2). The self-assessed significance of the specific use of color Doppler was also to be documented (Table 3). Recordings of all these examinations were to be stored as clinical routine exams in the local digital image archive and databases, assuring that they would be available for future review. The time used to achieve the proper projections of the fetal heart was also noted in the protocol at each examination.

**Table 2:** Self- assessed confidence to perform the method in clinical praxis

1	Uncertain
2	More uncertain than certain
3	More certain than uncertain
4	Certain

**Table 3:** The Significance of color Doppler for assessment of the heart anatomy

1	No significance
2	Little significance
3	Great significance
4	Crucial

The recordings from the group discussions were transcribed and analyzed generating a number of preliminary themes. These themes then served as a basis for the formation of the interview guide for the individual interviews. After approximately three months training with the new technique, individual interviews were performed on site with each midwife. The interview guide is shown in Table 4. The interviews were recorded and transcribed word for word. The interview texts were then analyzed qualitatively, using a simple content analysis to describe the midwives’ reflections on the use of what was for them a new technique. The method of content-analysis was inspired by the methodology described by Graneheim et al. [21]. The unit of analysis was the midwives’ experiences of working with the new technique. Meaning units regarding the challenges were marked in the transcript. These were then sorted into three content areas; ‘color’, ‘orientation’, and ‘hands-on’. Representative manifest quotations were chosen to illustrate the experienced challenges.

**Table 4:** Interview guide for the individual interviews

**Introduction**	**Questions**
During the course you had the opportunity to try color Doppler.	How did you think the color-Doppler helped you to see deviations?
	How did the color affect your ability to see deviations from normal?
	How can color be used to facilitate the assessment?
	How would you compare the assessment without color and with color added?
Several of the course participants said that assessment is facilitated if they themselves perform the ultrasound examination rather than assessing images obtained by someone else.	What do you think about that?
	Is it possible to “see” with your hands? How?
During the course you were to decide whether a number of cases were normal or abnormal.	What is important for you to be able to make a good assessment?
	What do you need to be able to judge an image as normal or not?
	What do you do if you are unsure?
Several of the course participants found it difficult to make the judgment with the help of images.	What do you find that is difficult about this?
	If you look at your ability to make judgments now, compared with before the course, how has it been affected?
	How have you been able to train your ability to make judgments after the course?
If you look at the structure of the course after completing it	What do you think about that now?
	What could have been done differently and why?
If you compare your ability to use the technique now compared to before the course.	What do you think about that now?

**Figure 1:**
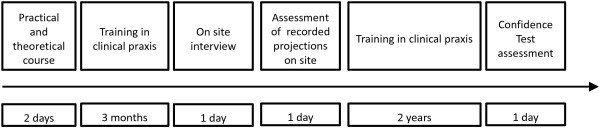
Flowchart of the learning program.

The ultrasound recordings from the first and last 10 exams during this three month period were reviewed and assessed by a specialist in fetal medicine and by a cardiac sonographer specialist. They graded the ability of the midwife to perform the different steps in the examination. The flow of the training program is presented in Figure 1.

Two years after the primary course, another follow up was performed. The same midwives documented 10 ultrasound examinations performed in the clinical routine in a way similar to the procedure followed during the training program. The follow up included the self-assessment concerning confidence in using the method (Table 2) and an identical review of the ultrasound recordings performed by the same specialists. Additionally, the midwives took a test identical to the pre-course test described above, where a series of ultrasound recordings were assessed; Dictaphones were not used, however.

The Regional Ethical Review Board in Linköping has approved the study (EPN 7–2009).

## Results

### Results from the 2-day initial course

The results at the pre- and post-course test were similar, 23/132 vs 21/132 incorrect answers (ns). The self-assessed uncertainty was, however, lower at the post-course test; 30/132 vs 11/132 (p = 0.006).

### Qualitative evaluation of the think-aloud recordings, group discussions and interviews

The group discussions prior to the training showed that the use of color Doppler produced unfamiliar images that some of the midwives found difficult to interpret. *‘It was all blurred, colors all over the place.’* This difficulty was also commented on in the think-aloud protocols of the pre-course test. The training seems to have enhanced the familiarity with the new technique; one of the midwives commented on the learning ‘*you could not kind of recognize where you were, at first, when you did not see the usual image…but you learn to kind of change your gaze, I believe…’* The importance of the hands-on training was emphasized as being critical for learning. The judgment of the scans only, as in the pre-course test, seemed, however, to deprive the midwives of some of the manual skills in identifying anatomical landmarks needed to perform the ultrasound, *‘you have to do it yourself, to know exactly the positions where you are’.* This aspect was also commented on in the three months follow up interview *‘you know the position of the fetal heart..but you get a totally different perception of what is up and down, left and right, when you’re watching someone else doing it, when you have no idea of how they hold the probe..It is really difficult..*’ Table 5 illustrates the four midwives’ judgments of 33 scans in the pre- and after-course test. Each scan consisted of several views, making several judgments possible.

**Table 5:** Midwives’ judgments of 33 scans before and after the course

**Judgments**	**Before training**	**After training**
No judgment made	28	29
Judgment without motivation	17	13
Judgment with motivation based on >1 view	33	36

Several judgments possible per scan.

### Results from the 3 months fetal screenings

In total, 80 examinations (20 from each midwife) were performed with the new method in the screening-situation three months after the course. The results are presented in Table 6. Their median self-assessed ability to technically use the new method was 3 out of 4 and the importance of color Doppler was graded 2.5 out of 4. From their self-assessment it was seen that it was possible to perform a full examination as defined in the protocol in 56/77 (73%) cases for gray-scale ultrasound registrations and in 53/77 (69%) for color Doppler registrations (ns). Data on self-assessment of the ability to perform the different steps were missing in three patients. The median time used to obtain the proper projections of the heart was four minutes (range 3–10 min).

**Table 6:** Self-assessment of the examinations in clinical praxis, number of correct performed examinations when assessed by specialist in fetal medicine and specialized sonographer and time spent to achieve the proper projections

	**At three months**	**At two years**	**p-value**
Self-assessed confidence to perform the method, median/range	3/(1–4)	4/(2–4)	-
Self-assessed significance of color-Doppler, median/range	2.5/(2–4)	3/(1–4)	-
Self-assessed correctly performed examinations in grey scale, No/out of (%)	56/77 (73%)	33/40 (83%)	0.240
Self-assessed correctly performed examinations in color Doppler, No/out of (%)	53/77 (69%)	32/40 (80%)	0.199
Specialist review: Correctly performed gray-scale examinations, No/out of (%)	67/80 (84%)	30/40 (75%)	0.251
Specialist review: Correctly performed color-Doppler examinations, No/out of (%)	52/80 (65%)	33/40 (83%)	0.047
Time spent to achieve the proper projections median/range (min)	4/(3–10)	3/(2–5)	<0.01

From the specialist review of the 10 first and 10 last exams carried out by each midwife during the 3-month follow-up period with routine fetal screenings including the new method 67/80 (84%) grey scale and 52/80 (65%) color Doppler recordings were judged to be complete (ns). There were, however, large inter-individual differences in success-rate of correctly performed recordings ranging from 20/20 to 9/20 where the midwives with the longest experience had the best results.

### Results from the two-year follow-up

Two years after the course the same midwives recorded 40 new ultrasound examinations of the fetal heart including the new method, 10 by each midwife. Their median self-assessed ability to technically use the new method was 4 of 4 and the importance of color Doppler was graded 3 out of 4. From their self-assessment it was seen that it was possible to perform a full examination as defined by the protocol in 33/40 (83%) cases for gray-scale and 32/40 (80%) cases for color Doppler (ns).

From the specialist review of the 40 examinations 30/40 (75%) gray scale and 33/40 (83%) color Doppler recordings were judged to be complete (Table 6).

Results from the written test were in accordance with the results from the pre- and after-course tests (ns). Nineteen out of 132 answers were incorrect corresponding to a self-assessed uncertainty in 17 out of 132 exams.

The median time used to achieve the proper projections of the fetal heart and color-Doppler recordings was 3 minutes (range 2–5 min). This was significantly shorter than at the three month follow-up.

## Discussion

In the present study we describe the self-estimated difficulties and show the ability of the midwives to successfully add new ultrasound modalities to a standardized fetal ultrasound screening of the heart. By designing a short initial course based on a combination of the experience and actual needs and skills of the participants, hands-on training as advocated reduced uncertainty, but it was found that continuous on-site training with clinical and technical back-up are important.

In the present screening program in the South Eastern region of Sweden the fetal heart is considered normal if it is possible to obtain and verify a normal four-chamber view. This has, however, been proven to be highly ineffective for the detection of most CHD [5,6]. Is it possible, with a short course and further on-site training with backup, to achieve a better detection rate in a routine clinical screening situation in a low-risk population in Sweden? As noted in the introduction, the antenatal detection of CHD could be significantly increased by adding the three-vessel view [7,14,16]. McBrien et al. used a relatively simple training program to improve the detection rate for CHD from 28% pre-training to 43% in the year of training. The program included hands-on training and a refresher day where the sonographers were given a new lecture on fetal echocardiography and cases with CHD were reviewed, emphasizing the importance of continuous and repeated training [8]. Pézard et al. studied the difference between sonographers attending a training course and those not attending. The sensitivity vas 37% versus 16% respectively for detecting a CHD, which further strengthens the importance of continuous training [11]. However, the size of that study was small and the methods of training not specified. The possibility to use color-Doppler for additional screening for flow abnormalities in the heart seems appealing. In the new guidelines from the International Society of Ultrasound in Obstetrics and Gynecology (ISUOG) it is stated that the outflow tract and the four chamber view as a part of the screening is evidence-based. The use of color Doppler is not considered mandatory in the second trimester screening, but its use is, however, encouraged [22]. It has mainly been used to achieve additional information about already detected CHD, in particular complex cases and its use for screening is controversial [17,18]. Eggebo et al. found color-Doppler useful in the screening situation in Norway where 9/26 findings of CHD were related to the additional information given by the color.

In the present study we found that the midwives’ ability to examine the fetal heart could be improved, and that their level of self-confidence could be heightened. We also found that this could be done in the normal screening program without creating time-delays. A simple 2-day course in the technique was good enough to help them begin to use the technique in standard clinical praxis, however there were inter-individual differences. These were judged to depend on the clinical experience level of the midwife although the number of participants did not allow for statistical evaluation. One possible explanation of the differences was that it was difficult to make the correct settings in the equipment and this caused the cineloops to be too short. The midwives in our study were able to acquire sufficient skills to perform the different views, especially the part in gray-scale. They found it was more difficult to perform the color-Doppler registrations, and the midwives also considered the color-Doppler to be less significant in the assessment of the heart anatomy, a view that may change with time and experience [23,24]. At the follow-up after two years in practice, the ability to perform the examination was in accordance with the results from the 3-month follow-up, suggesting that the level of skill in performing the different steps in the examination persists if it is used in every-day practice. They still considered it to be difficult to perform Color-Doppler after two years in the self-assessment, but improved when analyzing the ability to obtain the correct views. The time spent to achieve the proper projections was also shorter, indicating that use in clinical practice improves the skills in obtaining the views wanted and that it does not significantly increase the time needed to administer the exam. The results from the written test given on three occasions did not show any improvements in interpreting images. One might conclude that assessment of the images obtained with the method is a more useful method to evaluate the introduction of a new way of performing the examinations than a written test based on review of acquired ultrasound images.

The combination of qualitative and quantitative analysis used is here a strength. The qualitative methods used were content analyses of participants “think aloud” notes/protocols during a pre- and post-course test, an audio-recorded group discussion session, and the recorded interviews about using the new technique, including the self-assessment of the performance to assess the fetal heart in clinical practice. One possible improvement of this study might have been to video record the pre-and post-course test for subsequent analysis. Reviewing the recordings together with the midwives would have given them the opportunity to comment on their own way to think during the test. This might have provided us with more data for analysis. The amount of data from the interviews was also limited. A specialist in interviewing technique might have provided more data. A deeper analysis was thus not possible. Therefore a simple content analysis was performed. The quantitative methods in the study were assessment of the ultrasound recordings by specialists including number of complete recordings and the time used for obtaining the images.

In this small study with only four participants the authors contributed to the course, methods of follow up, interpretation of data and analysis of the results, which might have influenced the results. The differences in the test result before and after the course are small. There is, however, a tendency for the number of correct judgments (without giving reasons for how the judgment was reached) to decrease after training. A tendency is also seen that the number of correct judgments increases when based on one or more views.

To our knowledge this is the first time that the development of ways of reasoning during a practical course in ultrasound methodology has been described. Our experience suggests that the midwives learn, by using the new technique, to use more views as a basis for their judgments. To learn something, the learner has to discern the critical aspects of the object of learning. A condition for learning is that the learner gets to experience a variation in a dimension of that aspect, through potential alternatives [25]. The design of the training exposed the learners to a broad range of fetal heart types, which provides one of necessary conditions for learning. The midwives’ reflections immediately after the training and after three months underscore the importance of hands-on training to be able to connect and integrate the experience based knowledge and skills with the new technology.

The size of the present study is a limitation but the results support the hypothesis that it is possible to add new aspects of an examination in the screening program by providing a relatively short training course. Additionally, repeated training with feed-back is needed to maintain the manual skills, obtained through experiential learning in every-day clinical practice.

## Conclusions

By participating in a continuous well-structured training program the competence of the midwives could become sufficient, without significant time increase, to perform a more accurate fetal cardiac ultrasound screening by adding five additional transverse views and color-Doppler. If this method is used in the screening situation it might increase the detection of fetal cardiac defects and thereby ensure the optimal care for the affected children after birth. These encouraging results must, however, be further evaluated by including a larger number of midwives and a long term follow up of CHD detection rates.

## Competing interests

The authors declare that they have no competing interests.

## Authors’ contributions

All authors contributed to the course, methods of follow up, interpretation of data and analysis of the results. EH, MAD and BJS planned the first protocol, EH and CS performed the assessment of the recorded ultrasound images. CS performed the interviews with the participating midwives. MAD and EH performed the qualitative evaluation of the interviews. EH and MAD drafted the original manuscript and all authors contributed in the revisions and final approval of the submitted manuscript.

## Pre-publication history

The pre-publication history for this paper can be accessed here:

http://www.biomedcentral.com/1471-2342/14/20/prepub
